# Identification and functional verification of active volatiles recognition by the olfactory co-receptor Orco in *Chrysopa pallens* (Rambur) (Neuroptera: Chrysopidae)

**DOI:** 10.1093/jisesa/ieaf036

**Published:** 2025-09-30

**Authors:** Shuangjiao Li, Xuyuan Gao, Chao Ma, Zhenya Tian, Yan Zhang, Hongsong Chen, Zhongshi Zhou

**Affiliations:** State Key Laboratory for Biology of Plant Diseases and Insect Pests, Institute of Plant Protection, Chinese Academy of Agricultural Sciences, Beijing, China; National Nanfan Research Institute, Chinese Academy of Agricultural Sciences, Sanya, China; National Nanfan Research Institute, Chinese Academy of Agricultural Sciences, Sanya, China; Guangxi Key Laboratory for Biology of Crop Diseases and Insect Pests, Institute of Plant Protection, Guangxi Academy of Agricultural Sciences, Nanning, China; State Key Laboratory for Biology of Plant Diseases and Insect Pests, Institute of Plant Protection, Chinese Academy of Agricultural Sciences, Beijing, China; National Nanfan Research Institute, Chinese Academy of Agricultural Sciences, Sanya, China; State Key Laboratory for Biology of Plant Diseases and Insect Pests, Institute of Plant Protection, Chinese Academy of Agricultural Sciences, Beijing, China; National Nanfan Research Institute, Chinese Academy of Agricultural Sciences, Sanya, China; State Key Laboratory for Biology of Plant Diseases and Insect Pests, Institute of Plant Protection, Chinese Academy of Agricultural Sciences, Beijing, China; National Nanfan Research Institute, Chinese Academy of Agricultural Sciences, Sanya, China; National Nanfan Research Institute, Chinese Academy of Agricultural Sciences, Sanya, China; Guangxi Key Laboratory for Biology of Crop Diseases and Insect Pests, Institute of Plant Protection, Guangxi Academy of Agricultural Sciences, Nanning, China; State Key Laboratory for Biology of Plant Diseases and Insect Pests, Institute of Plant Protection, Chinese Academy of Agricultural Sciences, Beijing, China; National Nanfan Research Institute, Chinese Academy of Agricultural Sciences, Sanya, China

**Keywords:** *Chrysopa pallens* (Rambur), olfactory recognition, function of olfactory receptor co-receptor, electroantennogram, RNA interference

## Abstract

Insect olfactory receptor co-receptor (Orco) plays a key role in olfactory organ formation and odor recognition during insect growth and development. In predatory insects, the olfactory system is important for the orientation, identification, and selection of hosts or prey. The cloning of the *Orco* gene in *Chrysopa pallens* (Rambur) holds significant importance, enabling evolutionary comparisons of olfactory systems among Neuroptera insects and opening possibilities for developing *Orco*-based biological control strategies through the manipulation of odor perception. The olfactory recognition mechanisms of the lacewing, *C. pallens* (Rambur), are poorly understood. One of the more significant findings to emerge from this study is that we cloned and sequenced the full-length *Orco* gene (*CpalOrco*) from *C. pallens.* It has been demonstrated that *CpalOrco* is highly conserved and similar to Orco genes of coleopterous insects. Real-time quantitative PCR analysis showed that *CpalOrco* was mainly expressed in *C. pallens* antenna. Compared to the control group injected with dsRNA targeting enhanced green fluorescent protein (dsEGFP), RNA interference-mediated silencing of *CpalOrco* through targeted double-stranded RNA (dsOrco) delivery resulted in significant suppression of gene expression. Experimental evidence from electroantennogram assays indicated that downregulation of *CpalOrco* transcripts substantially compromised the olfactory perception of nonanal in *C. pallens*. Overall, the current study has confirmed that *CpalOrco* may play an important role in the *C. pallens* odor recognition process.

## Introduction

Predatory insects play important roles in controlling the population of herbivorous insects in ecosystems. Use of natural predator insects is a safe and effective strategy to prevent and control agricultural pest insects, and such biological control methods are expected to continue to be developed in the future. The predatory insect, *Chrysopa pallens* (Rambur) is widely distributed in most provinces of China (Lin et al. 2007). *C. pallens* (Rambur) feeds mainly on the eggs and larvae of aphids, whiteflies, thrips, lepidopterous insects and other pests, and has great value in biological control (Huang et al. 1990, He et al. 2000, Liu et al. 2007, 2011, Tang et al. 2017). Although much is known about its predatory role, our understanding of the olfactory-related molecular mechanisms in *C. pallens* (Rambur) remains limited.

Odor recognition is an important sensory function in insects that has crucial roles in insect physiological and ecological activities, including those of natural predator insects. An earlier study reported the importance of insects’s olfactory system, in aiding insects find food, courtship, and protection (Reisenman et al. 2016). Insect recognition of external odors mainly relies on a specific combination of odorant molecules and olfactory nerves mediated by odorant receptors (ORs) (Gong et al. 2008, Zhan et al. 2018). ORs are an important part of the insect olfactory system and plays an important role in the molecular mechanism of insect chemical signal recognition. Functional OR units comprise conventional ORs and nonconventional olfactory co-receptor (Orco) molecules (Touhara and Vosshall 2009, Kaupp 2010, Vosshall and Hansson 2011). ORs and Orco are often co-expressed as heterodimers on sensory neuron membranes, where Orco functions to enhance the sensitivity of ORs in odor recognition (Larsson et al. 2005). In insects such as *Drosophila melanogaster*, Orco (olfactory receptor co-receptor) has been demonstrated to play an indispensable role in olfactory signal transduction by forming heteromeric complexes with ORs (Pitts et al. 2004, Larsson et al. 2005). Therefore, elucidating the functional role of *C. pallens* Orco provides a theoretical foundation for developing olfactory interference-based biological control strategies.

Here, the most obvious finding to emerge from this study is that we characterized the *Orco* gene in *C. pallens* and the relevance of *Orco* genes in different species is clearly supported by our findings. Further, we investigated the expression profiles of the *Orco* gene in various lacewing tissues, as well as the role of Orco in lacewing olfactory mechanisms.

## Materials and Methods

### Insect Rearing and Tissue Collection

*C. pallens* (Rambur) insects were provided from the Institute of Plant Protection, Beijing Academy of Agriculture and Forestry Sciences. Adult insects were kept in a greenhouse at 26 to 28  °C, relative humidity 40% to 60%, and photoperiod (light:dark) 14:10 h, using soybean aphid (Homoptera: Aphididae) as a host. Soybean aphids were gifted by the Biological Control Laboratory of the Institute of Plant Protection, Chinese Academy of Agricultural Sciences, and maintained on soybean (*Glycine max* L., variety Huayou No. 5) indoors for many generations to establish a stable population. The breeding conditions of soybean aphid are consistent with those of *C. pallens*.

Tissue samples of antennae, head, leg, thorax, abdomen, wing, and proboscis were dissected from a total of 120 adults (60 males and 60 females). Each tissue sample was collected as 1 experimental sample, promptly frozen in liquid nitrogen, and stored at −80 °C until ready for use.

### RNA Isolation and cDNA Synthesis

Total RNA samples were separately extracted from different tissues using TRIzol Reagent (Invitrogen, USA), following the manufacturer’s protocols, and treated with DNaseI (Invitrogen, Carlsbad, CA, USA). RNA quality was evaluated by 1.0% agarose gel electrophoresis and Nanodrop 2000 (OD 260 /OD 280 ranged from 1.80 to 2.10). The first strand of the complementary DNA (cDNA) was synthesized from 1 μg of total RNA using a first-strand cDNA synthesis kit (TransGen Biotech, Beijing, China), according to the manufacturer’s protocol. Synthesized cDNA samples were stored at −20 °C until use.

### Cloning of *Orco* Sequences from *C. pallens*

Primers were designed according to the *Orco* sequence (Unigene ID c48959_g1) in the *C. pallens* transcriptome database, using Primer Premier 5.0 software, as follows: CpalOrcoF, 5′-GAATCAAGCCAATAGTCAT-3′, CpalOrcoR, 5′-ATCTCCCACGAAGGTAA-3′; primers were synthesized (Shanghai Shenggong Company). Antennal tissue cDNA is used as a template for PCR amplification using DNA polymerase. PCR amplification was performed using the following conditions: a denaturing step at 95 °C for 5 min, followed by 32 cycles (94 °C for 30 s, 55 °C for 30 s, 72 °C for 1 min), and a final step of 10 min at 72 °C. The resulting 151-bp PCR product was purified and ligated into the pEASY-T3 vector (TransGen Biotech, China). After transformation with vector, Trans1-T1 Phage Resistant Chemically Component Cell (TransGen Biotech, Beijing, China) were plated on LB/agar containing ampicillin, isopropyl thio-β-D-galactosi-de and 5-bromo-4-chloro-3-indolyl-β-D-galactoside (Beijing Chemical Works, Beijing). White colonies were then grown in liquid LB/ampicillin and more than 12 independent clones were sequenced.

### Sequence Analysis

Translate the *Orco* sequence into the amino acid sequences using ExPASy (https://web.expasy.org/translate/). The online tools SignalP 5.0 (http://www.cbs.dtu.dk/services/SignalP/), TMHMM 2.0 (http://www.cbs.dtu.dk/services/TMHMM/), SWISS-MODEL (https://swissmodel.expasy.org/), NetPhos 3.1 (http://www.cbs.dtu.dk/services/NetPhos/), ProtParam (https://web.expasy.org/protparam/), and SMART 8 (http://smart.embl-heidelberg.de/) were used to predict and deduce signal peptide sequences, transmembrane (TM) domain locations, tertiary structure, phosphorylation sites, molecular weight and isoelectric point, and conserved domains, respectively, in the Orco amino acid sequence (Blom et al. 1999, Krogh et al. 2001, Nielsen and Hedegaard 2017, Letunic and Bork 2017).

Orco sequences from other insect species were retrieved from the NCBI database, DNAMAN 7 (Lynnon Corportion, USA) software used for multiple sequence alignment. The phylogenetic tree was constructed utilizing the neighbor-joining (NJ) method, employing the P-distance model and pairwise deletion of gaps (Ye et al. 2006). This analysis was conducted with the MEGA 6.06 software package. The stability of the tree’s architecture was evaluated through a bootstrap analysis with 1,000 replicates. The amino acid sequences of all Orco genes employed in the sequence analysis are provided in Supplementary Data S1.

### *CpalOrco* Expression Profiles

The expression profiles of *CpalOrco* were analyzed using qRT-PCR. Total RNA was isolated from 7 different tissues, including male antennae (MA-An), female antennae (FA-An), head (H), thorax (T), abdomen (Ab), leg (L), and wing (W) obtained from the virgin adult beetles of both genders within 3 days of adult eclosion. The method of total RNA extraction was the same as described above. The concentration of each RNA sample wasstandardized to 1 μg/μl, and the cDNA was synthesized using a first-strand cDNA synthesis kit for qPCR (TransGen Biotech, Beijing, China) according to the manufacturer’s protocol. *CpalOrco* was used as the target gene, and GTP binding protein (GTP) (GenBank No. U15543) was as an internal standard (Li et al. 2013). qRT-PCR was performed using ABI 7500 (Thermo Scientific, Waltham, MA, USA) with TransStart Tip Top Green qPCR Supermix (TransGenBiotech,Beijing,China). The PCR reaction program was set as follows: 30 s at 94 °C and 40 cycles at 94 °C for 5 s and 60 °C for 34 s. The qRT-PCR primers were designed using Primer Premier 5.0 (PREMIER Biosoft International), and the efficiency of the primers was validated before gene expression analysis. Each qRT-PCR reaction was performed using 3 technical replicates and 3 biological replicates.

### RNAi Analysis of the *CpalOrco* Gene

The target sequence (nucleotides 324 to 474) of *CpalOrco* gene was amplified by RT-PCR using specific primers conjugated with 21 bases of a T7 RNA polymerase promoter. After PCR amplification using the primers, the obtained targeting fragment was used to synthesize dsOrco with a MEGAscript RNAi kit (Ambion, USA), according to the manufacturer’s instructions. As a control, we also synthesized dsRNA targeting enhanced green fluorescent protein (dsEGFP). Both dsOrco and dsEGFP were diluted to a concentration of 10 μg/μl and stored at −20 °C until they were needed.

Adults of *C. pallens* aged 2 to 3 d old were separated groups of 50 males and 50 females and subjected to RNAi microinjection. Before injection, an agarose plate was placed on an ice tray, and the *C. pallens* adults immobilized on the agarose plates. Then, 0.3 μl of dsOrco or dsEGFP (10 μg/μl) was injected in the pronotum of each insect using a PLI-100 Pico-Injector (Harvard Apparatus, Holliston, MA, USA), by manipulation under a microscope with an MP-255 Micromanipulator (Sutter, Novato, CA, USA). After 12 and 24 h of injection (with 50 male and 50 female adults in each treatment group), the antennae were removed using tweezers by gripping them at the base and then placed into Eppendorf tubes. The RNA samples were extracted and the mRNA was analyzed as previously mentioned. The primer sequences utilized for dsRNA synthesis can be found in Table 1.

**Table 1.** Oligonucleotides designed for RNAi and qRT-PCR analysis of *CpalOrco*

Unigene ID	Gene	Primer (5′–3′)
c48959_g1	*CpalOrco*	F: GAATCAAGCCAATAGTCAT R: ATCTCCCACGAAGGTAA
	*dsOrco*	F: TAATACGACTCACTATAGGGGCGGAAAGTGACGATGTT R: TAATACGACTCACTATAGGGTTGCTTGCTGCACGGAAT
	*EGFP*	F: ACATGAAGCAGCACGACTTCT R: GGCGCGGGTCTTGTAGTT
	*dsEGFP*	F: TAATACGACTCACTATAGGGTGAGCAAGGGCGAGGAG R: TAATACGACTCACTATAGGGCGGCGGTCACGAACTCCAG
	GTP	F: ATTGGAATGCTGTTGAGATTGAAG R: GCTCCAGGATCATGTTTGTCTA

### EAG Assay

The antennal responses of 60 male and female *C. pallens* with dsOrco-injected and dsEGFP-injected were recorded using the EAG assay. In *Ambrosia artemisiifolia*, over 50 chemical compounds have been identified. Based on previous behavioral tests of *C. pallens* in our laboratory, nonanal (Sigma-Aldrich) was selected for use in the EAG assay (Chalchat et al. 2004, Jones et al. 2005). The concentrations of nonanal were adjusted to 10 μg/μl by hexane, and hexane alone served as a blank control. Aspirate 10 μl of nonanal solution onto a filter paper strip (Whatman No. 1; length: 5 cm, width: 0.5 cm), placed on a Pasteur pipette (length, 20 cm) and used as the odor cartridge. At 12 and 24 h after dsRNA injection, adult insect heads were dissected with a precision surgical blade. Subsequently, the tips of the antennae are severed, creating small circular openings. To conduct EAG recording, a glass capillary reference electrode filled with a solution of 0.1 M KCl was affixed to the back of the insect heads. Additionally, a similar recording electrode was attached to the excised tip of the adult antennae. Activated carbon-filtered air through the antenna was humidified and constant flow (400 ml/min). During the odor stimulation, a controlled airflow of 300 ml/min was directed into the main airflow for a duration of 0.2 s. To ensure accurate measurement, hexane was utilized as a stimulant at the start and end of experiments to correct the EAG response for each antenna. Following each nonanal stimulation, a 30-s interval was provided to allow the antennae to recover. The analogue signal was detected through a probe and processed with a data acquisition controller (IDAC-4, Syntech, the Netherlands). Data were analyzed using EAG 2000 software (Syntech, The Netherlands).

### Data Analysis

Data from qRT-PCR and EAG assays were analyzed by using SPSS 22 software. The qRT-PCR data were assessed using the 2^–ΔΔCT^ method. Statistical significance was assessed by one-way ANOVA (Dependent Variable: relative gene expression levels; Independent Variable: treatment groups) followed by an LSD multiple comparison test. The level of significance was set at a significance level of 0.05 (*P* < 0.05).

## Results

### Identification and Sequence Analysis of *CpalOrco*

**Fig. 1.**
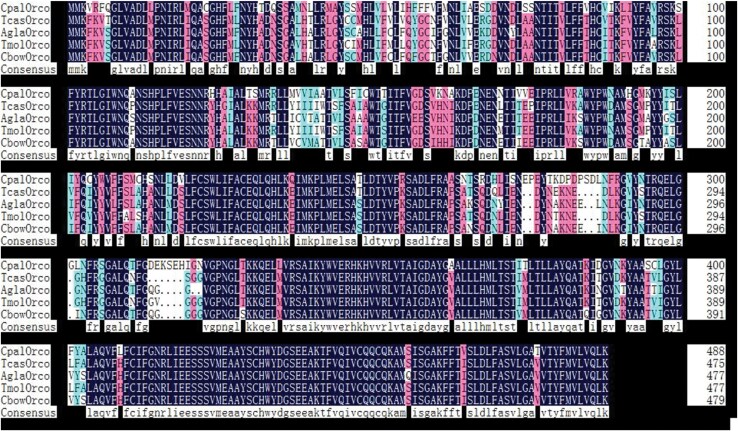
Alignment of the amino acid sequence encoded by CpalOrco with the other Orco from 4 coleopterous species. The identical sequences are marked with shading. CpalOrco: *Chrysopa pallens*; TcasOrco: *Tribolium castaneum* (GeneID:661975); AglaOrco: *Anoplophora glabripennis* (GeneID:108908594); TmolOrco: *Tenebrio molitor* (GeneID: 138128803); CbowOrco: *Colaphellus bowringi* (GeneID: 251636).

Utilizing a cDNA template in conjunction with designed PCR primers, the Orco gene from *C. pallens* was effectively cloned and designated as CpalOrco (Unigene ID: c48959_g1). The complete open reading frame (ORF) length was 1,467 bp, encoding 488 amino acids. The Smart online tool detected a 7TM-GPCR-Srsx domain at amino acid residues 36 to 185 of the *CpalOrco* sequence, while analysis with SignalP-5.0 did not identify any signal peptides in the protein. The estimated molecular weight of *CpalOrco*, determined using ProtParam, was 55.53 kDa, with an isoelectric point of 7.93. The amino acid sequence of *CpalOrco* exhibited 73.36% to 75.20% identity with Orco proteins from four coleopteran species: *Tribolium castaneum* (*TcasOrco*, 75.20%), *Anoplophora glabripennis* (*AglaOrco*, 73.36%), *Tenebrio molitor* (*TmolOrco*, 74.18%), and *Colaphellus bowringi* (*CbowOrco*, 74.80%). As illustrated in Fig. 1, the C-terminal domains were highly conserved among the 5 aligned sequences.

**Fig. 2.**
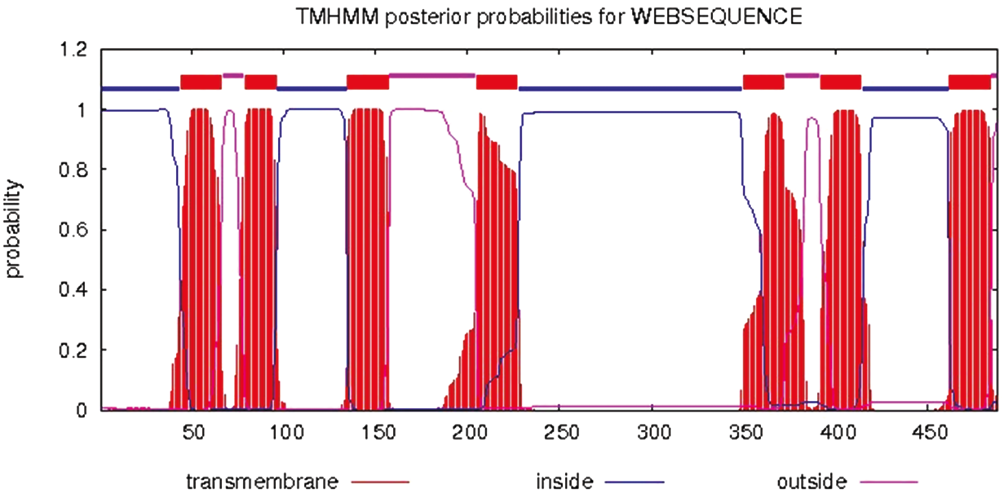
The TM structure of the *Chrysopa pallens* Orco protein predicted using TMHMM.

The TMHMM analysis indicated that CpalOrco contains 7 TM domains located at residues 44 to 66, 79 to 96, 135 to 157, 205 to 227, 350 to 372, 392 to 414, and 462 to 484, with distinct intracellular and extracellular loop regions as illustrated in the topology diagram (Fig. 2). The membrane topology of *CpalOrco*, with its N-terminus oriented intracellularly and C-terminus extracellularly, aligns precisely with the structural organization of typical insect olfactory receptors (Fig. 2).

**Fig. 3.**
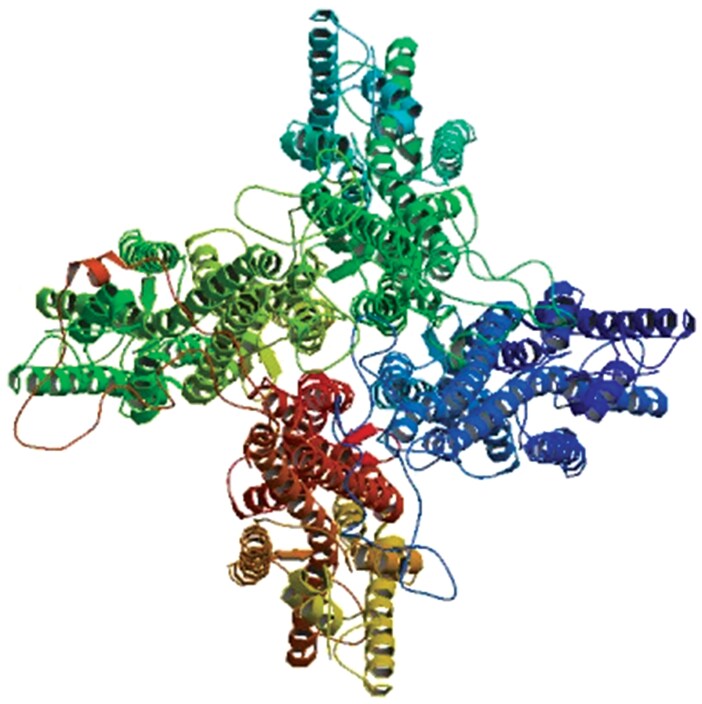
The tertiary structure of the *Chrysopa pallens* Orco protein predicted using Kad SWISS-MODEL. The figure represents the N-terminal, C-terminal, 7 TM domains, and the connecting intracellular and extracellular loops.

The tertiary structure of *CpalOrco* was predicted using SWISS-MODEL online, with the homologous Orco structure from Apis mellifera (PDB ID: 6C70) serving as the primary template. The model achieved a GMQE score of 0.74 and QMEAN score of −3.52, indicating moderate global quality despite expected limitations in loop region resolution. While the predicted tetrameric conformation aligns with conserved oligomerization patterns observed in insect Orco proteins, it should be noted that computational models primarily suggest thermodynamically favorable conformations rather than definitive in vivo states. To facilitate functional interpretation, we provide additional structural annotations in Fig. 3 showing monomeric topology with clearly demarcated N-terminal (residues 1 to 118), C-terminal (residues 372 to 409), and 7 characteristic TM domains connected by intracellular/extracellular loops (Fig. 3).

**Fig. 4.**
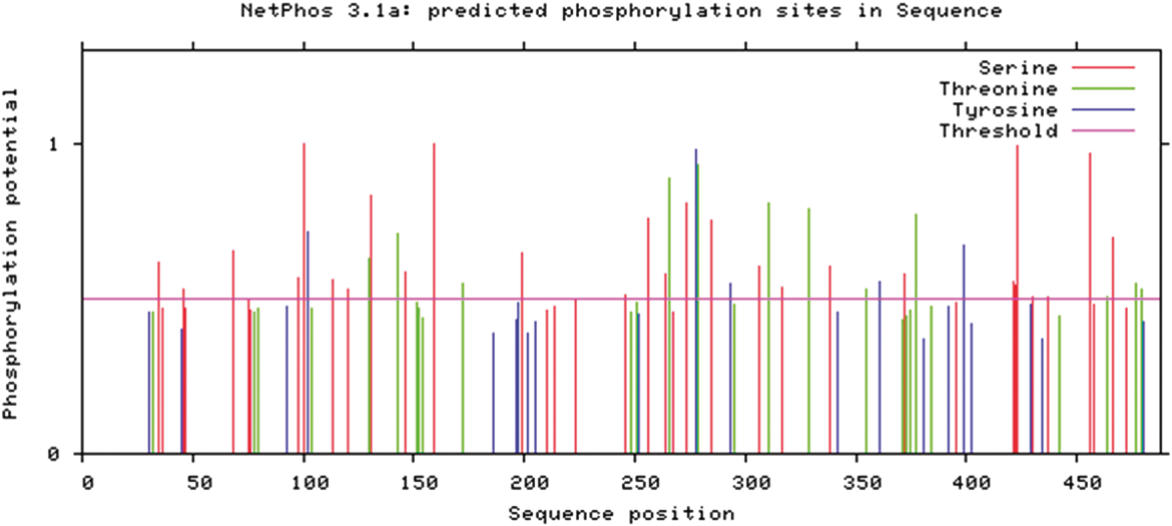
Phosphorylation sites in the *Chrysopa pallens* Orco protein predicted using NetPhos.

As protein phosphorylation has an important role in the physiological functions of organisms, we used the NetPhos online tool to predict phosphorylation sites in *C. pallens* Orco, revealing 40 serine (Ser), 16 threonine (Thr), and 8 tyrosine (Tyr) residues predicted to be phosphorylation sites (Fig. 4).

**Fig. 5.**
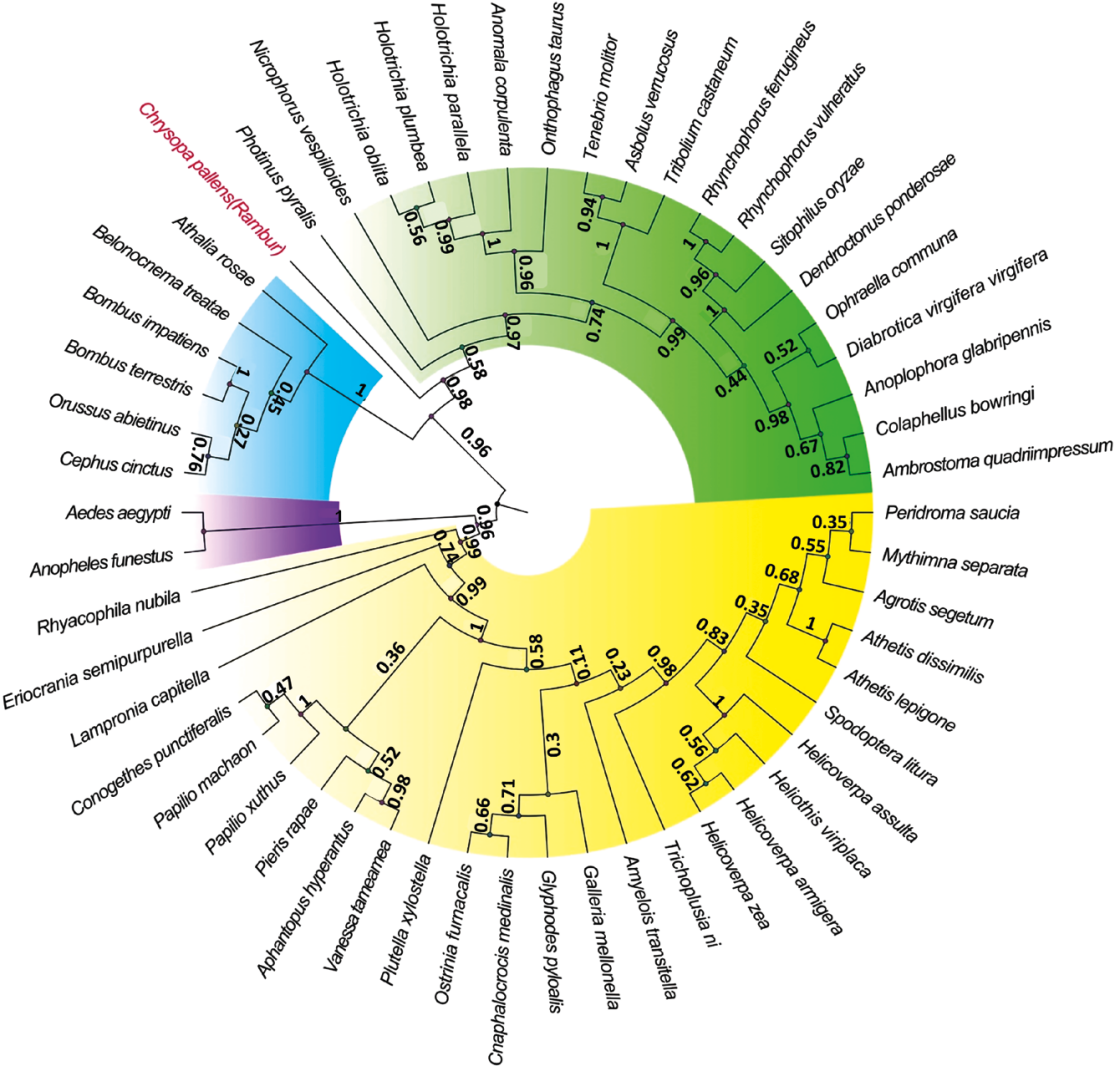
Neighbor-joining tree of Orco orthologues from different insects. The tree was constructed using MEGA6.06 with 1,000 iterations. Each branch is marked with a confidence value, and the branch lengths are proportional. The sequences of Orcos from other insects are shown in Supplementary Data S1.

Using MEGA6.06, we performed phylogenetic analysis of Orcos in insects from the orders, Neuroptera, Hymenoptera, Coleoptera, Diptera, and Lepidoptera. The Orco of each order of insects were clustered, and those of insects from different orders also exhibited a very high level of conservation. *CpalOrco* was most closely related to *Orco* sequences from coleopterous insects (Fig. 5).

### Expression Profile of *CpalOrco*

**Fig. 6.**
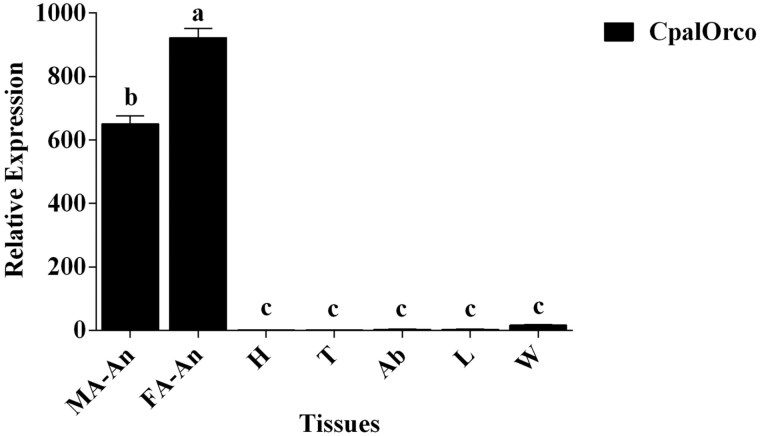
Expression profile in various tissues of *Chrysopa pallens* determined by qRT-PCR. The level of CpalOrco transcript was normalized relative to GTP. Each histogram bar represents the mean (±SD) from 3 repeats, and the different letters above the error bars indicate the significant difference by LSD test (*P* < 0.05). (MA-An: male antenna; FA-An: female antenna; H: head; T: thorax; Ab: abdomen; L: leg; W: wing).

To explore whether the *CpalOrco* candidate receptor gene is involved in insect olfaction, similar to other olfactory receptors, we examined its expression levels in various adult *C. pallens* tissues by qRT-PCR. We found that *CpalOrco* expression levels were highest in the antennae, and that there was a small amount of expression in the wings. Expression levels were highest in FA-An, followed by MA-An, and levels in both were significantly higher than those in other tissues (*F* = 737.41; df = 4; 20, *P* < 0.0001). There were no significant differences in the expression levels among the other tissues (Fig. 6).

### Biological Function of *CpalOrco*

**Fig. 7.**
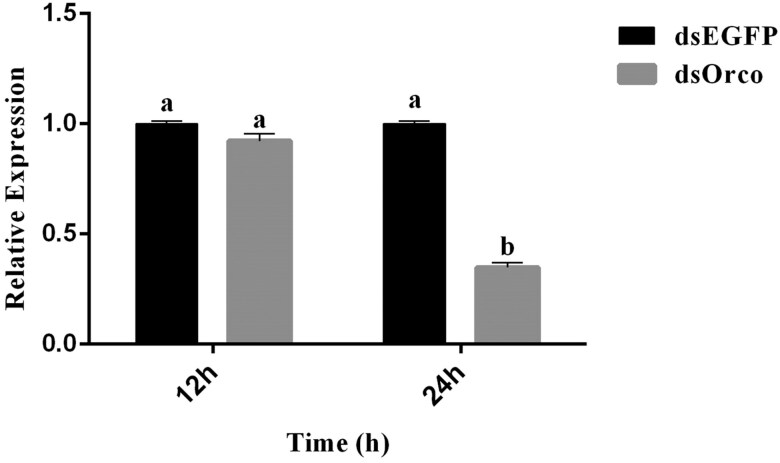
CpalOrco transcript levels in dsOrco- and dsEGFP-injected *Chrysopa pallens.* The different letters represent significant difference by one-way ANOVA and LSD test (*P* < 0.05).

*CpalOrco* knockdown was confirmed by qRT-PCR at 12 and 24 post-ds *CpalOrco* injection. At 12 h after injection, there was no significant difference between insects in the control (treated with RNAi targeting GFP injection) and dsOrco groups (*F* = 4.87; df = 1, 3; *P* = 0.09). While 24 h after injection, the expression of *CpalOrco* was significantly lower in the dsOrco RNAi-treated group than that of the dsEGFP-treated control group (*F* = 745.17; df = 1, 3; *P* < 0.0001). Further, *Orco* expression levels were significantly lower (66%) 24 h after injection of dsRNA than those 12 h after injection (Fig. 7).

**Fig. 8.**
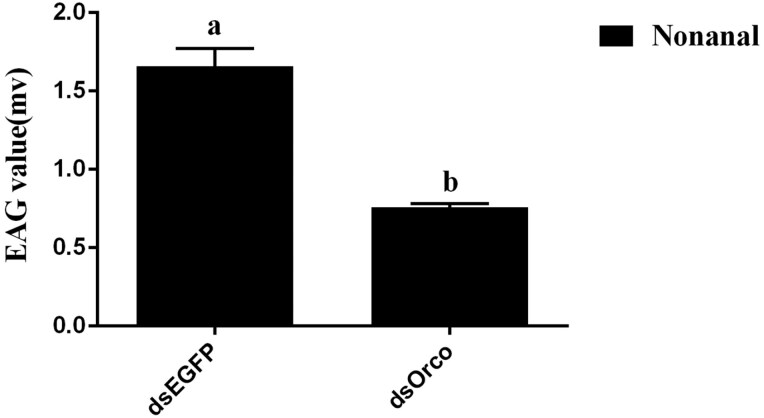
Electroantennogram analysis of RNAi-treated *Chrysopa pallens*. The different letters above the error bars indicate the significant difference by one-way ANOVA and LSD test (*P* < 0.05).

Next, EAG analysis was used to examine the antenna responses of dsEGFP- and dsOrco-treated insects. Insects the control group generated a significant antenna potential response to nonanal, while the potential change in response to nonanal was significantly lower after injection of dsOrco (*F* = 62.85; df = 1, 11; *P* < 0.0001) (Fig. 8).

## Discussion

In this study, several significant findings have emerged that enhance our understanding of insect olfactory systems. ORs convert chemical information in insect olfactory organs into electrical signals, and are considered central to insect peripheral olfactory reception. Orco is 1 of the 2 types of OR that does not participate directly in the identification of odorants, but can function as a nonselective cation channel. Further, Orco can improve the sensitivity of OR detection of odor molecules. Orco and ORs are generally expressed in the same sensory neuron membrane OR–Orco complexes form ligand-gated ion channels through the conserved Orco C-terminus and participate in insect olfactory responses. Hence, Orco has important roles in identifying various odors (Benton 2006, Sato et al. 2008, Wicher et al. 2008), as well as in reproductive physiology and social behavior plasticity (Yan et al. 2017). Our findings corroborate *CpalOrco* role in olfactory processing and highlight its potential as a strategic target for developing novel pest management strategies or conservation approaches through precise modulation of its activity in biological systems.

In this study, the *CpalOrco* cDNA was obtained by transcript sequencing, identifying a full length 1,467 bp nucleotide sequence encoding a total of 488 amino acids, which showed a high degree of conservation on multiple sequence alignment with Orco sequences from other insect species. Experimental data indicate that these Orco exhibit conserved functional roles across diverse insect species, as demonstrated in comparative analyses (Hill et al. 2002). The C-terminus of the Orco sequence of different insects is highly conserved, indicating that this region likely plays an indispensable role in the function of OR and Orco in ORs (Hopf et al. 2015, Yan et al. 2017). Further analysis of the phylogenetic relationship among Orco sequences revealed that *CpalOrco* is most closely related to those of coleopterous insects, with a longer genetic distance to those of lepidopteran insects. These findings are consistent with the results of Carmean et al. (1992), who demonstrated that the Coleoptera and Neuroptera are sister groups, and with the results of previous multiple sequence alignment analysis (Butterwick et al. 2018).

The *CpalOrco* protein was predicted to contain 7 TM structures, where the N-terminus is inside the membrane and the C-terminus is outside the membrane, identical to the membrane topology of other insect Orco proteins (Carmean et al. 1992, Wang 2013, Song et al. 2015). Further, the *CpalOrco* sequence was predicted to form a tetrameric structure, similar to the structure of *Apocrypta bakeri* Orco determined by cryo-electron microscopy (Zhou 2014).

Structural and functional studies reveal that site-specific dephosphorylation at position of the Orco phosphorylation domain regulates OR sensitivity through conformational changes in ligand-binding pockets (Poudel et al. 2021). Here, we predicted that the Orco protein contains 40 Ser, 16 Thr, and 8 Tyr phosphorylation sites, adding to understanding of the structure and mode of action of Orco.

The *CpalOrco* sequence information obtained provided a foundation for subsequent tissue expression and functional analysis. qRT-PCR analysis demonstrated that *CpalOrco* was mainly expressed in antennae, and that the expression levels were higher in females than males. This is consistent with results from analyses of *Culex pipiens pallens* and *Bactrocera dorsalis* (Zhao et al. 2013, Pitts et al. 2014, Liu et al. 2017), and suggest that the *Orco* gene has a primary olfactory function in the antennae. The differential expression detected between males and females may be related to the number, type, and perception of different chemical information substances, as well as differences in the functions of female and male antennae (Gao et al. 2020).

In this study, the gene silencing approach was used to interfere with the expression of *CpalOrco*, which was significantly decreased 24 h after injection of dsRNA, indicating successful silencing of *CpalOrco* at this time point. Further, the results of EAG assays showed that the potential change in response to nonanal significantly decrease after injection of dsRNA, indicating that Orco plays an important role in the process of olfactory recognition. In addition, recent studies have demonstrated that Orco also has important regulatory effects during insect wing differentiation, egg production, and life extension, among other physiological functions (Chu et al. 2007, Fan et al. 2015, Ma et al. 2020). In the future, further research is needed to explore the functions of Orco in other aspects of insect life.

In summary, this study used RNAi technology to conduct an initial exploration of *CpalOrco* function, providing a foundation for future experiments to determine the ligand specificity of *C. pallens* ORs and the molecular mechanisms involved in its signal transduction. Further, it provides a new possibility for using predatory insects to control invasive plants. In the future, we can manage pest populations and control the proliferation of invasive plant species by leveraging the response of *C. pallens* to specific odor compounds, so as to achieve the purpose of protecting the healthy growth of crops.

## Supplementary Material

ieaf036_suppl_Supplementary_Datas_S1
