# Supplementary material for: Identification and functional verification of active volatiles recognition by the olfactory co-receptor Orco in Chrysopa pallens (Rambur) (Neuroptera: Chrysopidae)
Source: J Insect Sci. 2025 Sep 30;25(4):8. doi: 10.1093/jisesa/ieaf036 (PMC12480744; doi:10.1093/jisesa/ieaf036)
Supplement: ieaf036_suppl_Supplementary_Datas_S1 [file ieaf036_suppl_supplementary_datas_s1.doc]

>c48959_g1 odorant receptor coreceptor [*Chrysopa pallens* (Rambur)]

MMKVRFQGLVADLLPNIRLMQACGHFLFNYHTDQSSAMNLLRMAYSSMHLVLVLIHFFFVFMNLIAESDDVNDLSSNTITILFFVHCVIKLIYFAVRSKSFYRTLGIWNQANSHPLFVESNNRHHAIALTSMRRLLMVVIAATVLSFICWTTITFVGDSVKNAKDPENENNTIVVEIPRLLVRAWYPWNAMHGMKYYISLIYQCYWVFFSMCHSNLLDVLFCSWLIFACEQLQHLKQIMKPLMELSATLDTYVPRSADLFRAASNTSRDHLISNEPEYTKDPDPSDLNFRGVYNTRQELGGLNFRSGALQTFGDEKSEHIGNVGPNGLTKKQELLVRSAIKYWVERHKHVVRLVTAIGDAYGAALLLHMLTSTITLTLLAYQATKIDGVNKYAASCLGYLFYALAQVFLFCIFGNRLIEESSSVMEAAYSCHWYDGSEEAKTFVQIVCQQCQKAMSISGAKFFTVSLDLFASVLGATVTYFMVLVQLK

>XP_008194693.1 PREDICTED: odorant receptor coreceptor [*Tribolium castaneum*]

MMKFKVTGLVADLMPNIRLIQASGHFMLNYHADNSGALHTLRLGYCCMHLVFVLVQYGCNFVNLVLERGDVNDLAANTITVLFFTHCVTKFVYFAVRSKLFYRTLGIWNQPNSHPLFVESNNRYHGIALKKMRRLLYIIIIWTSFSAIAWTGITFVGDSVHNIKDPENENLTITEPIPRLLVKAWYPWDAMSGMPYYITLVFQIYYVFFSLAHANLLDSLFCSWLIFACEQLQHLKEIMKPLMELSATLDTYVPKSADLFRAPSATSQDQLIENDYNEKNEDLKGVYSTRQELGGHFRGGALQNFGSGGVGPNGLTKKQELMVRSAIKYWVERHKHVVRLVTAIGDAYGVALLLHMLTSTIMLTLLAYQATKITGVDKYAATVLGYLLFALAQVFHFCIFGNRLIEESSSVMEAAYSCHWYDGSEEAKTFVQIVCQQCQKAMSISGAKFFTISLDLFASVLGAVVTYFMVLVQLK

>XP_018568191.1 odorant receptor coreceptor [*Anoplophora glabripennis*]

MMKFKVSGLVADLMPNIRLIQASGHFMFNYHADNSGALHALRLGYSCAHLLFCLFQYGCIFGNLVVEKDDVNYLAANTITVLFFTHCITKFVYFALRSKLFYRTLGIWNQSNSHPLFVESNNRYHALALKKMRTLLICVTATTVLSAAAWTGITFVEESVHNIKDPDNENETITEEIPRLLIKSWYPWDAMSGMAYYGSLIFQIYYVLFSLAHANLMDSLFCSWLIFACEQLQHLKEIMKPLMELSASLDTYVPKSADLFRAPSAKSQDNYIENDYNAKNEELNLKGIYNTRQELGGNFRSGALQTFGQGGVGPNGLTKKQELMVRSAIKYWVERHKHVVRLVTAIGDAYGVALLLHMLTSTVMLTLLAYQATKINGVNTYAATTIGYLVYSLAQVFHFCIFGNRLIEESSSVMEAAYSCHWYDGSEEAKTFVQIVCQQCQKAMQISGAKFFTISLDLFASVLGAVVTYFMVLVQLK

>AJO62219.1 olfactory co-receptor ORco [*Tenebrio molitor*]

MMKFKVSGLVADLMPNIRLIQASGHFMLNYHADNSGAVHTLRLGYCIMHLIFMLLQYGCNFVNLIFERGDVNDLAANTITVLFFTHCITKFVYFAARSKLFYRTLGIWNQPNSHPLFVESNNRYHALALKKMRRLLYIIIIWTSFSAIAWTSITFVGDSVHNIKDPDNENMTITEEIPRLLVKAWYPWNAMSGMPYYITLVFQVYYVFFALSHANLLDSLFCSWLIFACEQLQHLKEIMKPLMELSASLDTYVPKSADLFRAPSATSQDNLIENDYNTKNEDLKGVYSTRQELGGHFRGGALQNFGGVGGGVGPNGLTKKQELMVRSAIKYWVERHKHVVRLVTAIGDAYGVALLLHMLTSTIMLTLLAYQATKITGVDKYAATVIGYLLFALAQVFHFCIFGNRLIEESSSVMEAAYSCHWYDGSEEAKTFVQIVCQQCQKAMSISGAKFFTISLDLFASVLGAVVTYFMVLVQLK

>QEE83332.1 odorant co-receptor [*Ophraella communa*]

MMKFKVTGLVADLLPNIRLIQASGHFMFNYHADNSGSLHTLRVAYSCMHLIFCLFQYGCTFGNLVKEKDDVNYLAANTITVLFFSHCITKFVYFALRSKLFYRTLGIWNQPNSHPLFLESNNRYHALTLKKMRTLIVCVVAATVLSAAAWTGITFVGESVHNIKDPDNENDTIVEEIPRLLIKSWYPWNAMSGMTYYITLVFQIYYVLFSLMHANLLDSLFCSWLIFACEQLQHLKEIMKPLMELSASLDTYVPKSADLFRAPSANSQDNLIENDYNAKNDELNLKGIYNTRQEMGINFRSGALQTFGQGGGGVGPNGLTKKQELMVRSAIKYWVERHKHVVRLVTAIGDAYGVALLLHMLTSTVMLTLLAYQATKINGVNPYAASVIGYLVYALAQVFHFCIFGNRLIEESSSVMEAAYSCHWYDGSEEAKTFVQIVCQQCQKAMSISGAKFFTISLDLFASVLGAVVTYFMVLVQLK

>ALR72547.1 odorant receptor ORco [*Colaphellus bowringi*]

MMKFKVSGLVADLMPNIRLIQASGHFMFNYHADNSGALHALRLGYSCMHLVFCLFQFGCTFGNLVVERDNVNDLAANTITVLFFTHCITKFVYFAVRSKLFYRTLGIWNQANSHPLFVESNNRYHALALKKMRTLLVCVMATTVLSASAWTGITFVGDSIHHIKDPDNENETIIEEIPRLLVKSWYPWDAMSGTAYYASLIFQIYYVFFSLAHANLMDSLFCSWLIFACEQLQHLKEIMKPLMELSASLDTYVPKSADLFRAPSANSQDNLIENDYNAKNEEINLKGIYNTRQELGINFRSGALQTFGQGGGGVGPNGLSKKQELMVRSAIKYWVERHKHVVRLVTAIGDAYGVALLLHMLTSTVMLTLLAYQATQIGGVNKYAATVIGYLVYSLAQVFHFCIFGNRLIEESSSVMEAAYSCHWYDGSEEAKTFVQIVCQQCQKAMSISGAKFFTISLDLFASVLGAVVTYFMVLVQLK

>AJF94638.2 odorant receptor co-receptor [*Ambrostoma quadriimpressum*]

MMKFKVSGLVADLMPNIRLIQASGHFMFNYHADNSGALHALRLGYSCLHLVLCLVQFGCTFGNLVIERNDVNDLAANTITVLFFTHCITKFVYFAVRSKLFYRTLGIWNKANSHPLFLDSNNRYHALSLKKMRTLLICVMTTTILSASAWTAITFVGDSVHNVKDPDNDNETITEEIPRLLIKSWYPWNAMSGTAYYVSVSFQIYYVFFSLAHSNLMDSLFCSWLIFACEQLQHLKEIMKPLMELSASLDTYVPKSADLFRAPSANSQDNLIENEYNEKNEGLNLKGVYNTRQEMGANFRSGALQTFGQGGGGVGPNGLSKKQELMVRSAIKYWVERHKHVVRLVTAIGDAYGVALLLHMLTATVMLTLLAYQATKIDGVNKYAATVIGYLVYSLAQVFHFCIFGNRLIEESSSVMEAAYSCHWYDGSEEAKTFAQIICQQCQKALSISGAKFFTISLDLFASVLGAVVTYFMVLVQLK

>XP_017785109.1 PREDICTED: odorant receptor coreceptor [*Nicrophorus vespilloides*]

MMEFKTQGLVTDLMPNIRLMQASGHFLFNYHADNSGALHTLRIFYSCVHLILILLQFGCIFGNLVQEADDVNDLAANTITILFFTHCVTKFVYFAIRSKLFYRTLGIWNSPNSHPLFVESNNRYHSISLTKMRRVLMVVLATTLISAVSWTTLTFIGDSTHTKKDPNNENETITEEIPRLLVKAWYPWNAMSGMPYMLSLVYQVYYVLFSMLQSNLMDVLFCSWLIFACEQLKHLKAIMKPLMELSATLDTYVPKSADLFRANSAKSQDNLIENDYNNMKQDYKGVYNTQQEMGVNYRSGALQTFGPGGMNGGIGPNGLTKKQEMMVRSAIKYWVERHKHVVRLVTAIGDAYGVALLFHMLTSTITLTLLAYQATKIDGVTKYAASVIGYLLYALAQVFLFCIFGNQLIEESSSVMEAAYSCHWYDGSEEAKTFVQIVCQQCQKALSISGAKFFTISLDLFASVLGATVTYFMVLVQLK

>AEG88961.1 odorant receptor Or83b [*Holotrichia parallela*]

MMQFKPQGLVADLMPNIKLMKFAGHFMLNYYAENSGAVHTLRLGFCFGHLFLMLLQFGFTFGNLVQESDDVNDLAANTITILFFTHCIVKFIYFGVRQKLFYRTLGIWNQSNSHPLFLESNNRYHQLALTKMRRLLIIVMVGTIGSWIAWTTITFLGDSVHTRKDPSNENETITEEIPRLLVRSWYPWDAMSGIPYYITLVYQVYYVGFSMLHSNLLDSLFCSWLIFACEQLQHLKEIMKPLMELSATLDTYVPKSADLFRAPSASSQDRLMDSDYNARNEDVHMKTMYSTHHEMGVTYRSGQLQDFSGGIGPNGLTKKQELMVRSAIKYWVERHKHVVRLVTAIGDAYGIALLLHTSASTITLTLLAYQATKIDGVNKYALTVLGYLFYALTQVFHFCIFGNRLIEESSSVMEAAYSCHWYDGSEEAKTFVQIVCQQCQKAMSISGAKFFTISLVLFASVLGATVTYFMVLVQLK

>XP_028137484.1 odorant receptor coreceptor [*Diabrotica virgifera virgifera*]

MMKFKVSGLVADLMPNIRLIQASGHFMFNYYADNSGSLHTLRVGYCCVHLVLCLLQYGCTFGNLVVEREDVNDLAANTITVLFFTHCITKFVYFALRSKLFYRTLGIWNQPNSHPLFIESNNRYHALALKKMRTLLICVVATTVLSAAAWTGITFVGESVHNVKDPNNANETIVEEIPRLLVKSWYPFNAMSGGAYFLSLGFQIYYVLFSLMHANLLDSLFCSWLIFACEQLQHLKEIMKPLMELSASLDTYVPKSADLFRASSATSQDNLIENDYNTKNEEINLKGVYNTRQEMGANFRSGALQTFGPGGGGVGPNGLTKKQELMVRSAIKYWVERHKHVVRLVTAIGDAYGVALLLHMLTSTVMLTLLAYQATKINGVNPYAATVIGYLVYALAQVFHFCIFGNRLIEESSSVMEAAYSCHWYDGSEEAKTFVQIVCQQCQKAMSISGAKFFTISLDLFASVLGAVVTYFMVLVQLK>RZC33009.1 odorant receptor coreceptor [*Asbolus verrucosus*]

MSPQMMKFKVSGLVADLMPNIRLIQTSGHFMLHYHADNSGALHTLRLGYCCAHLVFMLLQYGSNFVNLIFERGDVNDLAANTITVLFFTHCITKFVYFAVRSKLFYRTLGIWNQPNSHPLFVESNNRYHALALKKMRQLLYIIVIWTSFSAIAWTSITFVGDSVHNIKDPDNENETITEEIPRLLVKGWYPWNAMSGMPYYITLIFQIYYVFFSLAHANLLDSLFCSWLIFACEQLQHLKEIMKPLMELSASLDTYVPKSADLFRAPSATSQDNLIESDYNAKNEELKGVYSTRQELGGHFRGGALQNFGGGGVGPNGLTKKQELMVRSAIKYWVERHKHVVRLVTAIGDAYGVALLLHMLTSTIMLTLLAYQATKITGVDKYAATVLGYLLFALAQVFHFCIFGNRLIEESSSVMEAAYSCHWYDGSEEAKTFVQIVCQQCQKAMSISGAKFFTISLDLFASVLGAVVTYFMVLVQLK

>AEE69033.1 olfactory receptor Or83b [*Holotrichia oblita*]

MMKFKPQGLVADLMPNIKLMKFAGHFMLNYYAENSGAVHTLRLGFCFGHLFLMLLQFGFTFGNLVQESDDVNDLAANTITVLFFTHCIVKFIYFGVRQKLFYRTLGIWNQSNSHPLFLESNNRYHQLALTKMRRLLIIVMIGTIGSWIAWTTITFFGDSVHNRKDPNNENETITEEIPRLLIRSWYPWDAMSGIPYYVSLIYQIYYVGFSMLHSNLLDSLFCSWLIFACEQLQHLKEIMKPLMELSATLDTYVPKSADLFRAHSASSQDKLTESDYNARNEDAHMRAMYSTHQEMGVTYRSGQLQEFSSGGIGPNALTKKQELMVRSAIKYWVERHKHVVRLVTAIGDAYGIALLLHMLTSTITLTLLAYQATKIDGVNKYALTVLGYLFYALAQVFHFCIFGNRLIEESSSVMEAAYSCHWYDGSEEVKTFVQIVCQQCQKAMSISGAKFFTISLDLFASVLGATVTYFMVLVQLK

>XP_030747553.1 odorant receptor coreceptor [*Sitophilus oryzae*]

MMNTFKVTGLVADLMPNIRLIQASGHFMLNYHADNSGALHALRLGYPCVHLLFVLLQYGCIFGNLVVEKDNVNDLAANTITILFFTHCLTKFVYFAARSKLFYRTLGIWNQANSHPLFVESNNRYHALALKKMRNLLYIIMIGTIFSAGAWSGITFVGDSVHFVKDPNNENETIPEEIPRLLIKSWYPFNAMSGMSYYIALVFQIYYVLFSLLQANLLDSLFCSWLIFACEQLQHLKEIMKPLMELSASLDTYVPKSADLFKSPSATSHDNLIENDYNPKNDELNLKGVYSTRQELGNLNFRSGALQTFGQGGGGVGPNGLSKKQELMVRSAIKYWVERHKHVVRLVTAIGDAYGVALLLHMLTATIMLTLLAYEATKIDGVNVYAATTLGYLIYSLAQVFHFCIFGNRLIEESSSVMEAAYSCHWYDGSEEAKTFVQIVCQQCQKALSISGAKFFTISLDLFASVLGAVVTYFMVLVQLK

>XP_022907054.1 odorant receptor coreceptor [*Onthophagus taurus*]

MMNFKVTGLVADLMPNIRLMQASGHFMLNYYADNNGALHTLRLGYCFMHLFLVLLQYGFTFGNLVQESDDVNDLAANTITVLFFTHCLTKFVYFALRSKLFYRTLGIWNQANSHPLFAESNNRYHALALTKMRRVLAIVVIGTLASWIAWTTITFFGDSTHTRKDPNNENETITEEIPRLLIKSFYPWNAMSGMKYYISLSYQVYYVLFSMLHSNLLDVLFCCWLIFACEQLQHLKEIMKPLMELSATLDTYVPKSADLFRAPSVNSQDNLIDNDALDYNNMKNDELNLKGIYSTHQEMGINYRGGNLQQFDSGGGGIGPNGLTKKQELLVRSAIKYWVERHKHVVRLVTAIGDAYGIALLLHMLTATITLTLLAYQATKIDSVSKYALTVLGYLFYALAQVFLFCIFGNRLIEESSSVMEAAYSCHWYDGSEEAKTFVQIVCQQCQKAMSISGAKFFTISLDLFASVLGATVTYFMVLVQLK

>AKC58535.1 odorant co-receptor [*Anomala corpulenta*]

MMQFKPQGLVADLIPNIKLMQFSGHFMLNYYAETTGAVHTLRLGFCFGHLFLLLLQFGFTFGNLVQQSDDVNDLAANTITVLFFTHCITKFVYFAVRQKLFYRTLGIWNQSNSHPLFLESNNRYHQLALTKMRRLLIVIMIGTIGSWIAWTTITFFGDSVHTRKDPNNENETITEEVPRLLVRSWYPWDAMSGAAYYVSLVYQIYYVGFSMLHSNLLDSLFCSWLIFACEQLQHLKEIMKPLMELSATLDTYVPKSADLFRAPSASSQDNLVDSDYNQSNEDANLRNLYTTHQEMGVTYRSGNLQEFSSGGIGPNGLSKKQELMVRSAIKYWVERHKHVVRLVTAIGDAYGIALLLHMLTSTIMLTLLAYQATKIDGVNKYALTVIGYLLYALAQVFHFCIFGNRLIEESSSVMEAAYSCHWYDGSEEAKTFVQIVCQQCQKAMSISGAKFFTISLDLFASVLGATVTYFMVLVQLK

>AOO35283.1 olfactory co-receptor [*Rhynchophorus ferrugineus*]

MNTFKVAGLVADLMPNIRLIQASGHFMLNYHADNSGALHGLRLGYCCMHLLFVLLQFGCIFGNLVKEKDNVNDLAANTITILFFTHCLTKFVYFAVRSKLFYRTLGIWNQANSHPIFIESNNRYHALALKKMRNLLYIIMIGTIFSASAWTGITFMGDSVHYIKDPNNENETISEEIPRLLIKSWYPFDAMSGMPYYIALVFQVYYVLFSLLHANLLDSLFCSWLIFACEQLQHLKEIMKPLMELSASLDTYVPKSADLFKAPNSASSQDNLIENEYNSKNDELNLKGVYSTRQELGNLTFRSGALQTFGQGGGGVGPNGLTKKQELMVRSAIKYWVERHKHVVRLVTAIGDAYGVALLLHMLTATIMLTLLAYEATKIDGVNVYAATTIGYLLYSLAQVFHFCIFGNRLIEESSSVMEAAYSCHWYDGSEEAKTFVQIVCQQCQKALSISGAKFFTISLDLFASVLGAVVTYFMVLVQLK

>ADM35103.1 olfactory receptor Or83b [*Holotrichia plumbea*]

MMQFKPQGLVADLMPNINLMKFAGHFMLNYYSDNGGALHTLRLGFCFGHLFLMLVQFGFTFGNLVQQSDDVNDLAANTITVLFFTHCIVKFIYFGVRQKLFYRTLGIWNQSNSHPLFLESNNRYHQLALTKMRRLLIVVMIGTIGSWIAWTTITFFGDSVHTTKDPNNENETITEEVPRLLIRAWYPWDAMAGIPYYISLVYQIYYVGFSMLHSNLLDSLFCSWLIFASEQLQHLKEIMKPLMELSATLDTYVPKSADLFRAPSASSQDKLTESDYNARNEDAHMRAMYSTHQEMGVTYRSGQLQDFSSGGIGPNGLTKKQELMVRSAIKYWVERHKHVVRLVTAIGDAYGIALLLHMLTSTITLTLLAYQATKIDGVNKYALTVLGYLFYALAQVFHFCIFGNRLIEESSSVMEAAYSCHWYDGSEEAKTFVQIVCQQCQKAMSISGAKFFTISLDLFASVLGATVTHFMVLVQLK

>AOO35284.1 olfactory co-receptor [*Rhynchophorus vulneratus*]

MNTFKVAGLVADLMPNIRLIQASGHFMLNYHADNSGALHGLRLGYCCMHLLFVLLQFGCIFGNLVKEKDNVNDLAANTITILFFTHCLTKFVYFAVRSKLFYRTLGIWNQANSHPIFIESNNRYHVLALKKMRNLLYIIMIGTIFSASAWTGITFMGDSVHYIKDPNNENETISEEIPRLLIKSWYPFDAMSGMPYYIALVFQVYYVLFSLLHANLLDSLFCSWLIFACEQLQHLKEIMKPLMELSASLDTYVPKSADLFKAPNSASSQDNLIENEYNSKNDELNLKGVYSTRQELGNLTFRSGALQTFGQGGGGVGPNGLTKKQELMVRSAIKYWVERHKHVVRLVTAIGNAYGVALLLHMLTATIMLTLLAYEATKIDGVNVYAATTIGYLLYSLAQVFHFCIFGNRLIEESSSVMEAAYSCHWYDGSEEAKTFVQIVCQQCQKALSISGAKFFTISLDLFASVLGAVVTYFMVLVQLK

>XP_031331912.1 odorant receptor coreceptor [*Photinus pyralis*]

MMKFKKAGLVADLFPNIKLMQGVGHFLFNYHSETGGAVHGLRVLYSSVHLVLLLAQFGFTFGNLIVESDDVNDLAANTITVLFFTHTITKYIYFALRSKMFYRTLGIWNQANSHPLFVDSNNRYHSIALKKMRQLLVVIVSVTIFSTISWTTITFFGPSERIRKDPENENMTISTEIPRLLIKSWYPWNAMAGMAHIVSLVYQVYYIFFSMSQANLADSMFCSWLIFACEQLMHLKEILKPLMELSATLDTYHPKTADLFRAPSANSQTNLVDDDYNDKNNHNDELDLRGIYSTGQELGTHFRSSALQSFNAGGGGIGPNGLTKKQELMVRSAIKYWVERHKHVVRLVTAIGDTYGIALLLHMLTSTIMLTLLAYQATKIDGVNPYAFSVIGYLLYALGQVFHFCIFGNRLIEESSSVMEAAYSCHWYDGSEEAKTFVQIVCQQCQKAMSISGAKFFTVSLDLFASVLGAVVTYFMVLIQLK

>XP_019768125.1 PREDICTED: odorant receptor coreceptor [*Dendroctonus ponderosae*]

MINKFKVVGLVADLMPNIRLIQASGHFMFNYYADNSGSLHILRLGYCCMHLFFVLVQYGCIFGNLVKEKDNVSHLAANTITILFFTHCLSKFIYFAARSKLFYRTLGIWNQANSHPIFLESSNRYHALALKKMRSLLYIILFGTIFSASAWTAITFVGESVHFIKDPDNDNETITEEIPRLLIKSWYPFDAMSGMTYYVALVFQIYYVFFSLFQANLLDNLFCSWLIFACEQLQHLKEIMKPLMELSATLDTFVPKSADLFKSPGSATSQDHLIENDFNAKNDDLKGVYSTRQELGNLNFRSGALQTFGQGGGGVGPNGLTKKQELMVRSAIKYWVERHKHVVRLVTAIGDAYGVALLLHMLTATVMLTLLAYEATKIDGLNTYAATTLGYLLYSLAQVFHFCIFGNRLIEESSSVMEAAYSCHWYDGSEEAKTFVQIVCQQCQKSLFISGAKFFTISLDLFASVLGATVTYFMVLVQLK

>ATV96621.1 odorant receptor co-receptor [*Eriocrania semipurpurella*]

MMTKFKVQGLVADLMPNIRLMQMSGHFMFNYYAENGSMAVMMRKAYSITHLVLILVQFACMLANMAMHTDDVNELTANTITVLFFVHSIIKLVYFPLNSKNFYRTLGIWNQSNSHPLFAESNARYHQSALKQMRMLLYFVSGFTLVSVTAWTTITFFGESVRLAKDKETNETITEVVPRLMIKAFYPFDAMSGTMYYIAFVYQLYFLIFALMLANLSDVMFCSWLIFACEQLQHLKAIMKPLMELSASLDTYRPNTAELFRAPSAGSQNALISEKEEKSPDPVDLDIRGIYSTRQDFGMNMRGAGGGLQTFGDGNGANPNGLTRKQELLVRSAIKYWVERHKHVVRLVAAVGDTYGTALLFHMLVSTITLTLLAYQATKVNSVDVYAFTVIGYLVYTLAQVFHFCIFGNRLIEESSSVMEAAYSCHWYDGSEEAKTFVQIVCQQCQKAMSISGAKFFTVSLDLFASVLGAVVTYFMVLVQLK

>XP_012253637.1 odorant receptor coreceptor [*Athalia rosae*]

MMKYKQEGLVADLMPNIRIMQISGHFMFNYYNDAGGSSIKLFHQIYCVVHLVLILLQFGLCCVNLIQESGDVDDLTADTITILFFAHALIKLGYFAIRSKMFYRTFGIWNNPNSHPLFAESNARYHALALTKMRRLLMAVGITTILSVIAWTGITFVGDSVKTTVDKETNETITVEIPRLMLRSWYPFDASHGMAHAIVVGYQFYWLLITMVDSNMLDVLFCSWLLFACEQLQHLKQIMKPLMELSATLDTVVPHTNDLFKAGSTEHLRDNEPPPPPPPNELLDLDLRGIYSNRQDFTATFKSSGGITFNGGVGPNGLTKKQEMLVRSAIKYWVERHKHVVRLVTSIGDAYGVALLFHMLVSTVTLTLLAYQATKVHGINVYAASVIGYLLYTLGQVFLFCIFGNRLIEESSSVMEAAYSCHWYDGSEEAKTFVQIVCQQCQKAMTISGAKFFTVSLDLFASVLGAVVTYFMVLVQLN

>AYN64391.1 odorant receptor coreceptor [*Rhyacophila nubila*]

MMNKFKVHGLVADLMPNIVMMKAFGFFLFNYYADNGAISVLIRKIYSSIHLVLIMVQFGGIVANMALQAVDINELTANTITVLFFSHSIVKFVFFAVTSKNFYRTLGIWNQSNTHPLFAESDARYHALAVTKMRRLLYAVTLGSILTVVAWTTITFFGESVRLTKDRETNETITEEVPRLPLKSWYPFDAMGGTPYMGCFAYQIYWLFFALMHANLLDMMFCSWLIFACEQLQHLKSIMKPLMELSASLDTYRPNTGELFRASAGSQTQLISPDDMKSSESLDVDLRGIYNNRQDFGMNFRTGSTLQTFGNNGVNGANPNGLTKKQEMLVRSAIKYWVERHKHVVRLVAGIGDTYGTALLFHMLISTITLTLLAYQATKIAGIDVYALTVLGYLFYTLAQVFLFCIFGNRLIEESSSVMEAAYSCHWYDGSEEAKTFVQIVCQQCQKAMSISGAKFFTVSLDLFASVLGAVVTYFMVLVQLK

>NP_001310774.1 odorant receptor coreceptor [*Cephus cinctus*]

MMKFKQQGLVADLMPNIRHMQFSGHFMFNYYNDTGGSTKLFHTIYCSIHLFLILLQFGLCCVNLTLERADVDDLTANTITVLFFAHSIIKLAYFAVRSKLFYRTLGIWNNPNSHPLFAESNARYHAIALTKMRRLLAAVGAATILTVCAWTGITFVGDSVKKVTDPVTNETMTVEIPRLMLRSWYPYDASHGMAHVLTLIYQFYFLLITTMDANSLDVLFCSWLLFACEQLQHLKQIMKPLMELSATLDTVVPHTNELFKAGSTDHLRDTQGTQPMAPPPNENMLDMDLRGIYSNRQDFTATFRTAAGMNFNGGVGPNGLTKKQEMLVRSAIKYWVERHKHIVRLVTAIGDAYGVALLFHMLITTVSLTLLAYQATKVNTVDVYAATVIGYVLYTLGQVFLFCIFGNRLIEESSSVMEAAYSCHWYDGSEEAKTFVQIVCQQCQKAMSISGAKFFTVSLDLFASVLGAVVTYFMVLVQLK>AIO10777.1 odorant receptor co-receptor [*Anopheles funestus*]

MQVQPTKYVGLVADLMPNIRLMQASGHFLFRYVTGPILIRKVYSRWTLIMVLMQFFAILGNLASNADDVNELTANTITTLFFTHSVTKFIYFAVNSENFYRTLGIWNQTNSHPLFAESDARYHSIALAKMRKLLVLVMATTILSVVAWVTITFFGESVQNVFDKETNETYKVVIPRLPIKSWYPWNAMSGPAYIFSFIYQIYFLLFSMVQSNLADVMFCSWLLLACEQLQHLKGIMRPLMELSASLDTYRPNSAALFRAISAGSKSELIINEEKDPDVKDFDLSGIYSSKADWGAQFRAPSTLQTFDENGRNGNPNGLTRKQEMMVRSAIKYWVERHKHVVRLVSAIGDTYGPALLLHMLTSTIKLTLLAYQATKIDGVNVYGLTVIGYLCYALAQVFLFCIFGNRLIEESSSVMEAAYSCHWYDGSEEAKTFVQIVCQQCQKAMTISGAKFFTVSLDLFASVLGAVVTYFMVLVQLK

>NP_001345400.1 odorant receptor coreceptor [*Aedes aegypti*]

MNVQPTKYHGLVLDLMPNIRLMQGFGHFLFRYVNGPVLIRKLYSWWNLIMILLQYFAIMGNLVMNTGDVNELTANTITTLFFTHSVTKFIYVAVNSEHFYRTLGIWNQPNSHSLFAESDARYHSIALAKMRKLLVMVMVTTVLSVVAWITITFFGDSVKNVFDKETNETYTVEIPRLPIKAWYPWDAMSGVPYFFSFIYQAYFLLFSMCQANLADVMFCSWLLFTCEQLQHLKGIMRPLMELSATLDTYRPNSAALFRVASAGSKSELILNEEKDPDTKDFDLNGIYNSKADWGAQFRAPSTLQTFGDNGINGNPNGLTKKQELMVRSAIKYWVERHKHVVRLVSAIGETYGAALLLHMLTSTIKLTLLAYQATKIDALNVYGLTVIGYLVYALAQVFLFCIFGNRLIEESSSVMEAAYSCHWYDGSEEAKTFVQIVCQQCQKAMTISGAKFFTVSLDLFASVLGAVVTYFMVLVQLK>XP_026490757.1 odorant receptor coreceptor [*Vanessa tameamea*]

MMTKIKTQGLVSDLMPNIKLMQLAGHFLFNYHSDNSGMSSLLRKIYSSVHAIFIVVHYVCMAVNMAKYSDEVNELTANTITVLFFAHSIIKLAFFAITSKNFYRTLAVWNQSNSHPLFIESDARYHQLALTKMRRLLYFICGMTIFSVICWVTITFFGDSVRLLMDKETNETLTEPVPRLPLKAWYPFDAMSGTMYVVAFVFQIYWLLFSMAIANLMDVMFCSWLIFACEQLQHLKAIMKPLMELSASLDTYRPNTADLFKVSSSEKSEKIPDPIDMDIRGIYSTQQDFGMTLRGAGGRLQTFGQQNLNNPNGLSQKQEMLARSAIKYWVERHKHIVRLVSSIGDTYGTALLFHMLVSTITLTLLAYQATKVDGLNVYAFSTVGYLSYTLGQVFHFCIFGNRLIEESSSVMEAAYSCQWYDGSEEAKTFVQIVCQQCQKAMSISGAKFFTVSLDLFASVLGAVVTYFMVLVQLK

>XP_012273699.1 odorant receptor coreceptor [*Orussus abietinus*]

MMKFKQQGLVADLMPNIRIMQYTGHFMFNYYNDAGGSIKFIHKIFCSVHLFLILLQFALCGVNLLFESGDVDDLTANTITMLFFTHSVVKLLYFAVRSKLFYRTLGIWNNPNTHPLFAESNARYHAIALTKMRRLLFCVGAATILSALAWTGITFVGDSVKKSIDPVTNETVIVEIPRLMLRSWYPFDASHGIAHIMILIYQFYWLLFSLADANSLDVLFCSWLLFACEQLQHLKQIMKPLMELSATLDTVVPNSSELFKAGSADHLRENQGSELSAPPPTSDNMLDLDLRGIYSNRQDFTATFRPTAGMTFNGGVGPNGLTKKQEMLVRSAIKYWVERHKHVVRLVTAIGDAYGVALLLHMLTTTITLTLLAYQATKVNSVDVYAATVIGYLLYTLGQVFLFCIFGNRLIEESSSVMEAAYSCHWYDGSEEAKTFVQIVCQQCQKAMSISGAKFFTVSLDLFASVLGAVVTYFMVLVQLK

>XP_031764320.1 odorant receptor coreceptor [*Galleria mellonella*]

MMTKVKAQGLVTDLMPNIKLMQAAGHFLFNYHSDNSGMSMLLRKVYSSVHAVLIVVNYVCMAINMAQYSDEVNELTANTITVLFFAHSVIKLLFFALNSKSFYRTLAIWNQSNSHPLFTESDARYHQLALTKMRRLLYCICGVTVLSVASWVTLTFFGESVRFIANKETNETMTEPAPRLPLKAWYPFNTMSGTMYIAAFALQIYWLLFSMAIANLLDVMFCSWLIFACEQLQHLKAIMKPLMELSASLDTYRPNTAELFRASSTEKSEKVPDPVDLDIRGIYSTQQDFGITLRGAGGKLQTFGQPTPNNPNGLTQKQEMLARSAIKYWVERHKHVVRLVASIGDTYGTALLFHMLISTITLTLLAYQATKIDGVNVYAFSTLGYLTYTLGQVFHFCIFGNRLIEESSSVMEAAYSCQWYDGSEEAKTFVQIVCQQCQKAMSISGAKFFTVSLDLFASVLGAVVTYFMVLVQLK

>XP_033216466.1 odorant receptor coreceptor [*Belonocnema treatae*]

MMKFKQQGLVSDLLPNIRLMQICGHFMFNYYGEAKKFAHKIYCFVHLFLILIQFGCMAFNLVEESADVDDLTANTITMLFFTHSVVKVIYFAARSKLFYRTLAIWNNPNSHPLFAESNARYHSIALTKMRRLLFCVGAATILSVISWTGITFVGESVKHITDPVTNETMTVEIPRLMLRAWYPFNAQHGIKHILMLVYQFYFLLITMADANSLDVLFCSWLLFACEQLQHLKQIMKPLMELSATLDTVVPNSSELFKAGSAEHLRDNPSPSAGENNMLDLDVRGIYSNRQDFTATFRPTAGMTFNGGVGPNGLTKKQEMLVRSAIKYWVERHKHVVRLVTAVGDAYGVALLLHMLATTITLTLLAYQATKVSGVDVYAASTIGYLLYTLGQVFLFCIFGNRLIEESSSVMEAAYSCHWYDGSEEAKTFVQIVCQQCQKAMSISGAKFFTVSLDLFASVLGAVVTYFMVLVQLK

>ALT31679.1 odorant receptor co-receptor [*Cnaphalocrocis medinalis*]

MMTKVKAQGLVSDLMPNIKLMQAAGHFLFNYHSDNAGMSTLLRKIYASAHAVLIVIHYLCMAVNMAQYSEEVNELTANTITVLFFAHSVIKLLFFAINSKSFYRTLAVWNQSNSHPLFTESDARYQQLALTKMRRLLYFICGVTVLAVVSWITLTFFGESVRLIANKETNETLTEPAPRLPLKAWYPFDAMSGTMYVVAFVYQIYWLLFSMAMANLLDVMFCSWLIFACEQLQHLKAIMKPLMELSASLDTYRPNTAELFRASSTDKSEKVPDPVDMDIRGIYSTQQDFGMTLRGAGGRLQNFGTNGSNPNGLTQKQEMLARSAIKYWVERHKHVVRLVASIGDTYGTALLFHMLVSTITLTLLAYQATKINGINVYAFSTIGYLSYTLGQVFHFCIFGNRLIEESSSVMEAAYSCQWYDGSEEAKTFVQIVCQQCQKALSISGAKFFTVSLDLFASVLGAVVTYFMVLVQLK

>ABU45983.2 odorant receptor Or83b [*Helicoverpa assulta*]

MMTKVKAQGLVSDLMPNIKLMQMAGHFLFNYHSENAGMSNLLRKIYASTHAILIVIHYACMGINMAKYSDEVNELTANTITVLFFAHTIIKLAFFALNSKSFYRTLAVWNQSNSHPLFTESDARYHQIALTKMRRLLYFICGMTVLSVISWVTLTFFGESVRMVTNKETNETLTEVVPRLPLKAWYPFNAMSGTMYIVAFAFQVYWLLFSMAIANLMDVMFCSWLIFACEQLQHLKAIMKPLMELSASLDTYRPNTAELFRASSTEKSEKIPDTVDMDIRGIYSTQQDFGMTLRGAGGRLQNFGQQNPNPNGLTPKQEMLARSAIKYWVERHKHVVRLVASIGDTYGTALLFHMLVSTITLTLLAYQATKINGINVYAFSTIGYLSYTLGQVFHFCIFGNRLIEESSSVMEAAYSCQWYDGSEEAKTFVQIVCQQCQKAMSISGAKFFTVSLDLFASVLGAVVTYFMVLVQLK

>AQY16483.1 odorant receptor co-receptor, partial [*Peridroma saucia*]

MMTKVKAQGLVSDLMPNIKLMQAAGHFLFNYHSENAGMSNLLRKIYASTHAILIIIHFACMGVNMAQYSDEVNELTANTITVLFFTHTIIKLGFFALNSKSFYRTLAVWNQSNSHPLFTESDARYHQIALTKMRRLLYFICGMTCLSVVCWITLTFFGESVRLITNKETNETLTEVAPRLPLKAWYPFNAMSGTMYIVAFAFQVYWLLFSMAIANLMDVMFCSWLIFACEQLQHLKAIMKPLMELSASLDTYRPNTAELFRASSTEKSEKIPDAVDMDIRGIYSTQQDFGMTLRGAGGRLQNFGQQNSNPNGLTPKQEMLARSAIKYWVERHKHVVRLVASIGDTYGTALLFHMLVSTITLTLLAYQATKINGINVYAFSTIGYLSYTLGQVFHFCIFGNRLIEESSSVMEAAYSCQWYDGSEEAKTFVQIVCQQCQKAMSISGAKFFTVSLDLFASVLGAVVTYFMVLVQLK

>XP_026726946.1 odorant receptor coreceptor [*Trichoplusia ni*]

MMAKVKAQGLVSDLMPNIRLMQAAGHFLFNYHSDNSGMTMLLRKVYASVHAVLIVIHFACMGVNMAQYSEEVNELTANTITVLFFAHTIIKLVFFALNSKSFYRTLAVWNQSNSHPLFTESDARYHQIALTKMRRLLYFICGMTALSVVSWVTLTFFGESVRLITSKETNETLTEPAPRLPLKAWYPFNAMGGSMYIVAFVFQVYWLIFSMAIANLMDVMFCSWLIFACEQLQHLKAIMKPLMELSASLDTYRPNTAELFRASSTEKSEKIPDAVDMDIRGIYSTQQDFGMTLRGAGGRLQNFASQNNPNPNGLTPKQEMLARSAIKYWVERHKHVVRLVASIGDTYGTALLFHMLVSTITLTLLAYQATKINGINVYAFSTIGYLSYTLGQVFHFCIFGNRLIEESSSVMEAAYSCQWYDGSEEAKTFVQIVCQQCQKAMSISGAKFFTVSLDLFASVLGAVVTYFMVLVQLK

>XP_021195606.1 odorant receptor coreceptor [*Helicoverpa armigera*] MMTKVKAQGLVSDLMPNIKLMQMAGHFLFNYHSENAGMSNLLRKIYASTHAILIFIHYACMGINMAKYSDEVNELTANTITVLFFAHTIIKLAFFALNSKSFYRTLAVWNQSNSHPLFTESDARYHQIALTKMRRLLYFICGMTVLSVISWVTLTFFGESVRMVTNKETNETLTEVVPRLPLKAWYPFNAMSGTMYIVAFAFQVYWLLFSMAIANLMDVMFCSWLIFACEQLQHLKAIMKPLMELSASLDTYRPNTAELFRASSTEKSEKIPDTVDMDIRGIYSTQQDFGMTLRGAGGRLQNFGQQNPNPNGLTPKQEMLARSAIKYWVERHKHVVRLVASIGDTYGTALLFHMLVSTITLTLLAYQATKINGINVYAFSTIGYLSYTLGQVFHFCIFGNRLIEESSSVMEAAYSCQWYDGSEEAKTFVQIVCQQCQKAMSISGAKFFTVSLDLFASVLGAVVTYFMVLVQLK

>AFI25169.1 odorant receptor 83b [*Heliothis viriplaca*]

MMTKVKAQGLVSDLMPNIKLMQMAGHFLFNYHSENAGMSNLLRKIYASTHAILIFIHYACMGINMAKYSDEVNELTANTITVLFFAHTIIKLAFFALNSKSFYRTLAVWNQSNSHPLFTESDARYHQIALTKMRRLLYFICGMTVLSVISWVTLTFFGESVRMVTNKETNETLTEVVPRLPLKAWYPFNAMSGTMYIVAFAFQVYWLLFSMAIANLMDVMFCSWLIFACEQLQHLKAIMKPLMELSASLDTYRPNTAELFRASSTEKSEKIPDTVDMDIRGIYSTQQDFGMTLRGAGGRLQNFGQQNNNPNGLTPKQEMLARSAIKYWVERHKHVVRLVASIGDTYGTALLFHMLVSTITLTLLAYQATKINGINVYAFSTIGYLSYTLGQVFHFCIFGNRLIEESSSVMEAAYSCQWYDGSEEAKTFVQIVCQQCQKAMSISGAKFFTVSLDLFASVLGAVVTYFMVLVQLK

>AAX14773.1 odorant receptor Or83b [*Helicoverpa zea*]

MTKVKAQGLVSDLMPNIKLMQMAGHFLFNYHSENAGMSNLLRKIYASTHAILIFIHYACMGINMAKYSDEVNELTANTITVLFFAHTIIKLAFFALNSKSFYRTLAVWNQSNSHPLFTESDARYHQIALTKMRRLLYFICGMTVLSVISWVTLTFFGESVRMVTNKETNETLTEVVPRLPLKAWYPFNAMSGTMYIVAFAFQVYWLLFSMAIANLMDVMFCSWLIFACEQLQHLKAIMKPLMELSASLDTYRPNTAELFRASSTEKSEKIPDTVDMDIRGIYSTQQDFGMTLRGAGGRLQNFGQQNPNPNGLTPKQEMLARSAIKYWVERHKHVVRLVASIGDTYGTALLFHMLVSTITLTLLAYQATKINGINVYAFSTIGYLSYTLGQVFHFCIFGNRLIEESSSVMEAAYSCQWYDGSEEAKTFVQIVCQQCQKAMSISGAKFFTVSLDLFASVLGAVVTYFMVLVQLK

>XP_028178675.1 odorant receptor coreceptor [*Ostrinia furnacalis*]

MMTKVKAQGLVSDLMPNIKLMQAAGHFLFNYHSDNSGMTTLLRKVYSSVHAFLIVINYLCMAANMAQYSEEVNELTANTITVLFFAHSVIKMLFFAVNSKSFYRTLAVWNQSNSHPLFTESDARYHQLALTKMRRLLYFICGVTVLAVMSWITITFFGESVRMIANKETNETLTEPAPRLPLKTWYPFDAMSGTMYVVAFVYQVYWLFFSMAIANLMDVMFCSWLIFACEQLQHLKAIMKPLMELSASLDTYRPNTAELFRASSTEKSEKMPDTVDMDIRGIYSTQQDFGMTLRGAGGRLQNFGQPNPNNPNGLTQKQEMLARSAIKYWVERHKHVVRLVASIGDTYGTALLFHMLVSTITLTLLAYQATKINGINVYAFSTIGYLSYTLGQVFHFCIFGNRLIEESSSVMEAAYSCQWYDGSEEAKTFVQIVCQQCQKAMSISGAKFFTVSLDLFASVLGAVVTYFMVLVQLK

>XP_013167416.1 PREDICTED: odorant receptor coreceptor [*Papilio xuthus*]

MMTKIKTQGLVSDLMPNIKLMQMAGHFLFNYYPENAGMSILLRKIYASVHAFLIIIQYLCMMANMAQYSDEVNELTANTITVLFFAHSIIKLIFFAINSKSFYRTLAMWNQSNSHPLFTESDARYHQLALTKMRRLLYFICGMTVLSVVCWVTITFFGESVRLITNKETNETLTEPAPRLPVKAWYPFNAMSGTMYVVAFIFQVYWLLFSMAIANLLDVMFCSWLIFACEQLQHLKAIMKPLMELSASLDTYRPNTAELFRVSNTEKSEKIPDTVDLDIRGIYSTQQDFGMTARGAGGRLQTFGQPAPNNPNGLTQKQELLARSAIKYWVERHKHVVRLVASIGDTYGTALLFHMLVSTITLTLLAYQATKIDGLNVYAFSTIGYLSYTLGQVFHFCIFGNRLIEESSSVMEAAYSCQWYDGSEEAKTFVQIVCQQCQKAMSISGAKFFTVSLDLFASVLGAVVTYFMVLVQLK

>QEI49014.1 odorant receptor co-receptor [*Mythimna separata*]

MMTKVKAQGLVSDLMPNIKLMQAAGHFLFNYHSENAGMSNLLRKIYASTHAILIIVHFACMGINMAQYSDEVNELTANTITVLFFTHTIIKLGFFALNSKSFYRTLAVWNQSNSHPLFTESDARYHQIALTKMRRLLYFICGMTCLSVVTWITLTFFGESVRMITSKETNETLTEVVPRLPLKAWYPFNAMSGTMYIVAFAFQVYWLLFSMAIANLMDVMFCSWLIFACEQLQHLKAIMKPLMELSASLDTYRPNTAELFRASSTEKSEKIPDAVDMDIRGIYSTQQDFGMTLRGAGGRLQNFGQQNANPNGLTPKQEMLARSAIKYWVERHKHVVRLVASIGDTYGTALLFHMLVSTITLTLLAYQATKINGINVYAFSTIGYLSYTLGQVFHFCIFGNRLIEESSSVMEAAYSCQWYDGSEEAKTFVQIVCQQCQKAMSISGAKFFTVSLDLFASVLGAVVTYFMVLVQLK

>XP_022831582.1 odorant receptor coreceptor [*Spodoptera litura*]

MMTKVKAQGLVSDLMPNIKLMQAAGHFLFNYHAENGGMTGLLRKIYASTHAILITIHFACLGINMAQYSDEVNELTANTITVLFFTHTIIKLGFFALNSKSFYRTLAVWNQSNSHPLFTESDARYHQIALTKMRRLLYFICGMTVLSVVSWVTLTFFGESVRLITSKETNETLTEVAPRLPLKAWYPFNAMSGTTYIIAFAFQVYWLLFSMAIANLMDVMFCSWLIFACEQLQHLKAIMKPLMELSASLDTYRPNTAELFRASSTEKSEKIPDTVDMDIRGIYSTQQDFGMTLRGAGGRLQTFGQQNNNPNGLTPKQEMLARSAIKYWVERHKHVVRLVASIGDTYGTALLFHMLVSTITLTLLAYQATKINGINVYAFSTIGYLSYTLGQVFHFCIFGNRLIEESSSVMEAAYSCQWYDGSEEAKTFVQIVCQQCQKAMSISGAKFFTVSLDLFASVLGAVVTYFMVLVQLK

>XP_011558816.1 PREDICTED: odorant receptor coreceptor [*Plutella xylostella*]

MMNKVKAQGLVSDLMPNIKLMQMAGHFLFNYHEENGGMSMLLRKIYASVHAFLIVIHYLCMLLNMAQYSDDVNELTANTITVLFFAHTVIKLLYFAINSKSFYRTLAVWNQSNSHPLFTESDARYHQLALTKMRRLMYFICAVTVLSVISWVTLTFFGESVRFIPDKETNETLTEPAPRLPLKAWYPFDAMSGGMYIVAFAYQVYWLLFAMAIANLMDVMFCSWLLFACEQLQHLKAIMKPLMELSASLDTYRPNTAELFRANSADKEKVPDPVDMDIRGIYSTQHDFGMTLRGAGGRLQNFGGQQVNNPNGLTQKQEMLARSAIKYWVERHKHVVRLVASIGDTYGTALLFHMLVSTITLTLLAYQATKIDGLNVYAFSTIGYLSYTLGQVFHFCIFGNRLIEESSSVMEAAYSCQWYDGSEEAKTFVQIVCQQCQKAMSISGAKFFTVSLDLFASVLGAVVTYFMVLVQLK

>XP_014363049.1 PREDICTED: odorant receptor coreceptor [*Papilio machaon*]

MMTKIKTQGLVSDLMPNIKLMQMFGHFLFNYYPENGGMSTLLRKIYASVHAFLIIIQYLCMMANMAQYSDEVNELTANTITVLFFAHSIIKLIFFAFNSKSFYRTLAMWNQSNSHPLFTESDARYHQLALTKMRRLLYFIFGMTVLSVVCWVTITFFGESVRLITNKETNETLTEPAPRLPVKAWYPFNAMNGTMYVVAFIFQVYWLLFSMAIANLLDVMFCSWLIFACEQLQHLKAIMKPLMELSASLDTYRPNTAELFRVSNTEKSEKIPDTVDLDIRGIYSTQQDFGMTARGAGGRLQTFGQPAPNNPNGLTQKQELLARSAIKYWVERHKHVVRLVASIGDTYGTALLFHMLVSTITLTLLAYQATKIDGLNVYAFSTVGYLSYTLGQVFHFCIFGNRLIEESSSVMEAAYSCQWYDGSEEAKTFVQIVCQQCQKAMSISGAKFFTVSLDLFASVLGAVVTYFMVLVQLK

>NP_001299600.1 odorant receptor coreceptor [*Amyelois transitella*]

MINNKVKAQGLVSDLMPNIKLMQASGHFLFNYYSDNSGMSMLLRKIYSSVHAILIVINYVCMVVNMAQYSDEVNELTANTITVLFFAHTVIKLLFFALNSKSFYRTLAVWNQSNSHPLFTESDSRHHQLALTKIRRLLYFICSMTVFSVVSWVTLTFFGESVRLIANKETNETISEPAPRLPLKTWYPFDAMGGSMYIIAFAFQVYWLFFSMITANLMDVMFCSWLIFACEQLQHLKAIMKPLMELSASLDTYRPNTAELFRVSSTEKSEKVPDPVDMDIRGIYATQQDFGMTLRGAGGRLQTFGQQNNNPNGLSQKQEMLARSAIKYWVERHKHVVRLVTSIGDTYGTALLFHMLISTITLTLLAYQATKIDGINVYAFSTIGYLSYTLGQVFHFCVFGNQLIEESSSVMEAAYSCQWYDGSEEAKTFVQIVCQQCQKAMSISGAKFFTVSLDLFASVLGAVVTYFMVLVQLK

>QIJ45831.1 odorant receptor co-receptor [*Glyphodes pyloalis*]

MMTKVKAQGLVSDLMPNIKLMQAAAHFLFNYHADNSGMSTLLRKIYSSVHAFLIVINYLCMAVNMAQYSDEVNELTANTITVLFFAHSIIKLLFFAITSKSFYRTLAVWNQSNSHPLFTESDARYHQLSLTKMRRLLYFVCGVTVLSVISWVTLTFFGESVRLIANKETNETLTEPAPRLPLKAWYPFNAMSGTMYVVAFVYQVYWLLFAMAIANLMDVMFCSWLIFACEQLQHLKAIMKPLMELSASLDTYRPNTAELFRASSTEKSEKVPEPVDMDIRGIYSTQQDFGITLRGAGGRLQNFAGGNNPNGLTQKQEILARSAIKYWVERHKHVVRLVASIGDTYGTALLFHMLISTITLTLLAYQATKINGINVYAFSTIGYLSYTLGQVFHFCIFGNRLIEESSSVMEAAYSCQWYDGSEEAKTFVQIVCQQCQKAMSISGAKFFTVSLDLFASVLGAVVTYFMVLVQLK

>XP_022131026.1 odorant receptor coreceptor [*Pieris rapae*]

MLNKVKTQGLVSDLMPNIKLMQMAGHFLFNYHSENDGMTSLLRKVYASVHALLIVTQYLCMVANMAQYSDEVNELTANTITILFFAHSIIKLLFFAITSKNFYRTLAVWNQSNSHPLFTESDARYHQLALTKMRRLLYFISGMTMLSVVCWVTITFFGDSVRLILDKDTNETLTEPAPRLPVKAWYPFNAMSGTMYIVAFVFQVYWLFFSMMIANLLDVMFCSWLIFACEQLQHLKAIMKPLMELSASLDTYRPNTADLFRVSSSGNKYYLIPFTLFHSDKNEKEPLDLDIRGIYSTQTDFGITLRGAGGRLQTFGQPPNNPNGLTQKQEMLARSAIKYWVERHKHVVRLVTSIGDTYGTALLFHMLVSTITLTLLAYQATKINGLNVYAFTTIGYLSYTLGQVFHFCIFGNRLIEESSSVMEAAYSCQWYDGSEEAKTFVQIVCQQCQKAMSISGAKFFTVSLDLFASVLGAVVTYFMVLVQLK

>XP_034828070.1 odorant receptor coreceptor [*Aphantopus hyperantus*]

MMTKVKTQGLVSDLMPNIKLMQAVGHFLFNYHSENAGMSVLLRKVYSSVHAVLIVAHYLCMAVNMAKYSEEVNELTANTITVLFFAHSLIKLLFFAVTSKNFYRTLAVWNQSNSHPLFTESDARYHQLALTKMRRLLYFICGTTIFSVTAWVTITFFGDSVRFLVDKETNETLTEEVPRLPLKAWYPFNAMSGTMYIAAFAFQIYWLLFSMAIANLLDVMFCSWLIFACEQLQHLKAIMKPLMELSASLDTYRPNTAELFKVNSSEKSPITEKIPDPIDMDIRGIYSTQQDFGMTLRGAGGRLQTFGQNPNNPNGLSQKQEMLARSAIKYWVERHKHIVRLVSSIGDTYGTALLFHMLVSTITLTLLAYQATKINGLNVYAFTTIGYLSYTLGQVFHFCIFGNRLIEESSSVMEAAYSCQWYDGSEEAKTFVQIVCQQCQKAMSISGAKFFTVSLDLFASVLGAVVTYFMVLVQLK

>AWV67916.1 odorant receptor co-receptor [*Lampronia capitella*]

MMTKFKVQGLVSDLMPNIKLMQMSGHYIFNYYEENGGMSMLMRKAYSAMHTVLITVQFLSMLANMAMYSDEVNELTANTITVLFFAHTLIKQLIFAVTSTNFYRTLAVWNQSNSHPLFTESDARYHQLSLQKMRQLLYLMLSITGFTVVSWTTITFFGESVRFISDKETNETMTEPVPRLPLKAWYPFNAMGGGMYILAFVYQIYWLTFSLLQANLLDVLFCSWLIFACEQLQHLKAIMKPLMELSASLDTYRPGTAELFRANSAEKQEKVPDPVDLDIRGIYSTRQDFGMNLRGGGGGLQTFGQPTAGNPNGLTQKQEMLVRSAIKYWVERHKHVVRLVASIGDAYGTALLFHMLVSTITLTLLAYQATKVDGVNVYAFSTIGYLLYTLGQVFHFCIFGNRLIEESSSVMEAAYSCHWYDGSEEAKTFVQIVCQQCQKAMSISGAKFFTVSLDLFASVLGAVVTYFMVLVQLK

>AGS41440.1 odorant receptor co-receptor [*Agrotis segetum*]

MMTKVKAQGLGSDLLPNIKLMQAAGHFLFNYHSENAGMSNLLRKIYASTHAILITIHFGCMAVNMAQYSDEVNELTANTITVLFFTHTIIKLSFFALNSKSFYRTLAVWNQSNSHPLFTESDARYHQIALTKMRRLLYFICGMTCLSVVFWITLTFFGESVRLITNKETNETLTEPVPRLPLKAWYPFNAMSGTMYIVAFAFQVYWLLFSMAIANLMDVMFCSWLIFACEQLQHLKAIMKPLMELSASLDTYRPNTAELFRASSTEKSEKIPDAVDMDIRGIYSTQQDFGMTLRGAGGRLQNFGQQNSNPNGLTPKQEMLARSAIKYWVERHKHVVRLVASIGDTYGTALLFHMLVSTITLTLLAYQATKINGINVYAFSTIGYLSYTLGQVFHFCIFGNRLIEESSSVMEAAYSCQWYDGSEEAKTFVQIVCQQCQKAMSISGAKFFTVSLDLFASVLGAVVTYFMVLVQLK

>XP_003402775.1 odorant receptor coreceptor [*Bombus terrestris*]

MMKFKQQGLVADLMPNIRLMKATGHFMFNYYTDNSTKTIHRIFAVVHLILMLMQFGFCGINLIFEKEDVDDLTANTITMLFFTHSVVKVVYFAVRSKLFYRTLGIWNNPNSHPLFAESNSRYHQVAVRKMRILLLAVLVTTMLSAISWTSITFIGDSVKKVIDPITNETTYVEIPRLMLRSWYPYNASHGMAHILTLIFQFYWLVFCMADANLLDVLFCSWLLFACEQIQHLKNIMKPLMEFSATLDTVVPNSGDLFKAGSAEQPRDHDPLPPTTPTAPGENMLDMDLRGIYSNRTDFTATFRPTAGMTFNGSVGPNGLTKKQEMLVRSAIKYWVERHKHIVRLVTAIGDAYGVALLLHMLITTITLTLLAYQATKINAVDTYAASVIGYLLYSLGQVFMLCIFGNRLIEESSSVMEAAYSCHWYDGSEEAKTFVQIVCQQCQKAMSISGAKFFTVSLDLFASVLGAMVTYFMVLVQLK

>ALJ33155.1 ORCO [*Athetis dissimilis*]

MMTKVKAQGLVSDLMPNIKLMQAAGHFLFNYHSENAGMSNLLRKVYASTHAILIVVNFACMGINMAQYSDEVNELTANTITVLFFTHTIIKLVFFALNSKSFYRTLAVWNQSNSHPLFTESDARYHQIALTKMRRLLYFICGMTCLAVVSWITLTFFGESVRLITNKETNETLTEVAPRLPLKAWYPFNAMSGTMYMIAFGFQVYWLLFSMAIANLMDVMFCSWLIFACEQLQHLKAIMKPLMELSASLDTYRPNTAELFRASSTEKSEKIPEAVDVDIRGIYSTQQDFGMTLRGAGGRLQNFGQQNANPNGLTPKQEMLARSAIKYWVERHKHVVRLVASIGDTYGTALLFHMLVSTITLTLLAYQATKINGINVYAFSTIGYLSYTLGQVFHFCIFGNRLIEESSSVMEAAYSCQWYDGSEEAKTFVQIVCQQCQKAMSISGAKFFTVSLDLFASVLGAVVTYFMVLVQLK

>AGF29886.1 odorant co-receptor [*Conogethes punctiferalis*]

MMNKVKALGLVSDLMPNIKLMQAAGHFLFNYHSDNSGMAMLLRKIYASVHAFLIVIHYLCMAVNMAQYSEEVNELTANTITVLFFAHSVIKLLFFALNSKSFYRTLAVWNQSNSHPLFTESDARYHQLSLTKMRRLLYFICGVTVLAVVCWVTITFFGESVRMIANKETNETLTEPAPRLPLKAWYPFDAMGGTMYVVAFVFQVYFLFFSMAIANLMDVMFCSWLIFACEQLQHLKAIMKPLMELSASLDTYRPNTAELFRASSTEKSEKVPDPVDMDIRGIYSTQQDFGMTLRGAGGRLQNFGGNPTNNPNGLTQKHEMLARSAIKYWVERHKHVVRLVASIGDTYGTALLFHMLVSTITLTLLAYQATKINGINVYAFSTIGYLSYTLGQVFHFCIFGNRLIEESSSVMEAAYSCQWYDGSEEAKTFVQIVCQQCQKAMSISGAKFFTVSLDLFASVLGAVVTYFMVLVQLK

>AOE48007.1 putative odorant receptor ORco [*Athetis lepigone*]

MMTKVKTQGLVSDLMPNIRLMQAAGHFLFNYHSENAGMSNLLRKVYASTHAILIVINFACMGINMAQYSDEVNELTANTITVLFFTHTIIKLSFFALNSKSFYRTLAVWNQSNSHPLFTESDARYHQIALTKMRRLLYFICGMTCLAVVSWITLTFFGESVRLITNKETNETLTEVAPRLPLKAWYPFNAMSGTMYMIAFGFQVYWLLFSMAIANLMDVMFCSWLIFACEQLQHLKAIMKPLMELSASLDTYRPNTAELFRASSTEKSEKIPEAVDVDIRGIYSTQQDFGMTLRGAGGRLQNFGQQNANPNGLTPKQEMLARSAIKYWVERHKHVVRLVASIGDTYGTALLFHMLVSTITLTLLAYQATKINGINVYAFSTIGYLSYTLGQVFHFCIFGNRLIEESSSVMEAAYSCQWYDGSEEAKTFVQIVCQQCQKAMSISGAKFFTVSLDLFASVLGAVVTYFMVLVQLK

>XP_003494153.1 odorant receptor coreceptor [*Bombus impatiens*]

MMKFKQQGLVADLMPNIRLMKATGHFLFNYYTDNSTKNIHRIFAIVHLVLMLMQFGFCGINLFFEKEDVDDLTANTITMLFFTHSVIKVVYFAVRSKLFYRTLGIWNNPNSHPLFAESNARYHQVAVRKMRILLLAVLATTMLSAISWTSITFIGDSVKKVIDPITNETTYVEIPRLMLRSWYPYNASHGMAHILTLIFQFYWLVFCMADANLLDVLFCSWLLFACEQIQHLKNIMKPLMEFSATLDTVVPNSGELFKSGSAEQPRDHDPLPPTTPTAPGENMLDMDLRGIYSNRTDFTATFRPTAGMTFNGGVGPNGLTKKQEMLVRSAIKYWVERHKHIVRLVTAIGDAYGVALLLHMLITTITLTLLAYQATKINAVDTYAASVIGYLLYSLGQVFMLCIFGNRLIEESSSVMEAAYSCHWYDGSEEAKTFVQIVCQQCQKAMSISGAKFFTVSLDLFASVLGAMVTYFMVLVQLK
